# Prospective Evaluation of All-lesion Versus Single-lesion Radiotherapy in Combination With PD-1/PD-L1 Immune Checkpoint Inhibitors

**DOI:** 10.3389/fonc.2020.576643

**Published:** 2020-10-29

**Authors:** Philipp Schubert, Sandra Rutzner, Markus Eckstein, Benjamin Frey, Claudia Schweizer, Marlen Haderlein, Sebastian Lettmaier, Sabine Semrau, Antoniu-Oreste Gostian, Jian-Guo Zhou, Udo S. Gaipl, Rainer Fietkau, Markus Hecht

**Affiliations:** ^1^ Department of Radiation Oncology, Universitätsklinikum Erlangen, Friedrich-Alexander-Universität Erlangen-Nürnberg, Erlangen, Germany; ^2^ Comprehensive Cancer Center Erlangen-EMN, Erlangen, Germany; ^3^ Institute of Pathology, Universitätsklinikum Erlangen, Friedrich-Alexander-Universität Erlangen-Nürnberg, Erlangen, Germany; ^4^ Department of Otolaryngology—Head & Neck Surgery, Universitätsklinikum Erlangen, Friedrich-Alexander-Universität Erlangen-Nürnberg, Erlangen, Germany; ^5^ Department of Oncology, The Second Affiliated Hospital of Zunyi Medical University, Zunyi, China

**Keywords:** radiotherapy, immunotherapy, immune checkpoint inhibitors, oligometastatic, single-lesion, all-lesion

## Abstract

**Background:**

Local ablative treatments improve survival in patients with oligometastatic disease in addition to chemotherapy. The application of immune checkpoint inhibitors prolonged patients’ survival in different tumor entities. This raises the question if patients still benefit from intensified local treatments in combination with a more efficient systemic treatment with immune checkpoint inhibitors.

**Methods:**

The prospective non-interventional ST-ICI trial investigates treatment with PD-1/PD-L1 (Programmed cell death protein 1/Programmed cell death 1 ligand 1) immune checkpoint inhibitors and radiotherapy in different tumor entities. Patients who started radiotherapy and immunotherapy concomitantly were included in this interim analysis. In this cohort patients with all-lesion radiotherapy (all tumor lesions irradiated, al-RT) were compared to patients with radiotherapy to only a single of their tumor lesions (single-lesion radiotherapy, sl-RT). Endpoints of the interim analysis were progression-free survival (PFS), overall survival (OS) and time to progression (TTP).

**Results:**

A total of 104 patients were registered between April 2017 and August 2019. Fifty patients started immune checkpoint inhibitor treatment and radiotherapy concomitantly and were included. Most frequent tumor entities were non-small cell lung cancer (62%) followed by head and neck squamous cell cancer (26%). Most frequent location of radiotherapy was lung (34%) and central nervous system (20%). Median duration of follow-up was 8.6 months beginning with first administration of the immune-checkpoint-inhibitor. Median PFS was 9.2 months (95% CI, 5.8 – 12.6) in the al-RT group and 3.0 months (95% CI, 2.5 – 3.5) in the sl-RT group (p<0.001). Median OS was 11.6 months (95% CI, 8.1 - 15.1) in the al-RT group and 4.2 months (95% CI, 3.0 - 5.4) in the sl-RT group (p=0.007). Median TTP was not reached in the al-RT group compared to 4.6 months (95% CI, 1.1–8.0) in the sl-RT group (p=0.028). Univariate Cox regression analyses computed tumor entity, histology, central nervous system metastases, immunotherapy drug and al-RT as predictors of OS (with an effect p-value of ≤ 0.1). In the multivariable analysis only tumor entity and al-RT remained prognostic factors for OS.

**Conclusion:**

Patients with PD-1/PD-L1 immune checkpoint inhibitor therapy benefit from local radiotherapy to all known lesions compared to single-lesion radiotherapy regarding PFS and OS.

## Introduction

Multi-disciplinary treatment strategies combining local ablative treatments and systemic therapy in patients with limited number of distant metastases, i.e. oligometastatic disease, improved survival in different tumor entities (1, 2). Especially in lung cancer, the combination of local ablative therapy in addition to chemotherapy improved survival (3). During the last years immune checkpoint inhibitors have improved survival compared to classical chemotherapy in several types of metastatic cancer and became first-line treatment (4, 5). However, most data about local ablative treatments in oligometastatic patients origin from the pre-immunotherapy era. Consequently, there is lack of evidence, whether patients still benefit from local treatments if more effective systemic treatment with immune checkpoint inhibitors is available (1, 6).

However, the combination of immune checkpoint inhibitors with radiotherapy might even be more efficient due to local immune-modulatory effects of radiotherapy (7). The expression of PD-L1 increases on the patients’ tumor cells after radiotherapy, which is the most important predictive parameter for anti-PD(L)1 inhibitors (8, 9). In preclinical models local radiotherapy in combination with immune checkpoint inhibitors also enhanced immunological effects distant to the irradiated area (10, 11). These systemic immune-modulating effects of local radiotherapy were also found in several clinical retrospective analyses (12–14).

The ST-ICI study was originally conducted to determine the effect of radiotherapy in combination with programmed cell death 1 (PD-1) or PD-L1 immune checkpoint inhibitors. In the current analysis, patients in an oligometastatic situation receiving radiotherapy to all known lesions (al-RT group) were compared to patients with radiotherapy to a fraction of known metastases (sl-RT group) in order to improve current treatment strategies.

## Material and Methods

### Trial Design and Treatments

ST-ICI is a prospective non-interventional, non-randomized, single-center trial investigating interactions of radiotherapy and immune checkpoint inhibitors. Patients with metastatic non-melanoma solid tumors of several entities and clinical indication for PD-L1/CTLA-4 therapy along with planed palliative radiotherapy according to clinical standard were included in the study. Patients that are known for substance abuse, not willing to take contraceptive measures, deemed uncooperative, not speaking German language and patients under legal care were not included in the trial. The current exploratory interim analysis focuses on patients with radiotherapy to all present metastases(al-RT) compared to patients receiving radiotherapy only to single of their metastases (single-lesion radiotherapy, sl-RT). The treatment decision was made by treating physicians based on clinical standards and national guidelines. Patients were allocated to the al-RT group, when local radiotherapy to all tumor lesions was possible according to the treating physician. Patients with single symptomatic metastases beyond other metastases received radiotherapy within the sl-RT group. Treatment with any EMA-approved inhibitor PD-1 or PD-L1 was allowed. Dosing and treatment indication of the immune checkpoint inhibitors was according to the European Medicines Agency (EMA) marketing authorizations. Radiotherapy was administered as stereotactic radiosurgery (SRS), stereotactic body radiotherapy (SBRT) or volumetric modulated arc radiotherapy (VMAT). The described total dose as well as dose per fraction was prescribed according local guidelines derived from national and international recommendations.

### Patients

Patients were eligible for this interim analysis if they were treated with immune checkpoint inhibitors and radiotherapy. Radiotherapy had to be delivered within a timeframe of ±30 days from the first administration of the immune checkpoint inhibitor. There was no limitation regarding tumor entity. As the trial should represent unselected patients, there were no limitations regarding baseline Eastern Cooperative Oncology Group (ECOG) performance status, pre-existing diseases, tumor entity or baseline blood parameters.

### Endpoint and Assessment

The objective in this exploratory interim analysis was to evaluate the efficacy of immune checkpoint inhibitors combined with al-RT compared to sl-RT. The endpoints of this interim analysis were overall survival (OS), progression-free survival (PFS) and time to progression (TTP). Survival analyses were deﬁned from the date of ﬁrst administration of immune checkpoint inhibitor, to the date of last follow-up, tumor progression or death. Data was collected from patients’ electronic health records (EHR) as well as the radiotherapy planning software (Pinnacle, Philips, USA). Survival data was provided by the Comprehensive Cancer Center Erlangen-EMN (CCC, Friedrich-Alexander University Erlangen-Nuremberg, Erlangen, Germany). Tumor staging was performed with computed tomography (CT) and/or magnetic resonance (MRI) imaging according to clinical standards.

### Trial Oversight

The ST-ICI trial was registered with ClinicalTrials.gov (identifier: NCT03453892). The institutional review board at the Friedrich-Alexander-Universität Erlangen-Nürnberg approved the study (number: 2_17 B). The study was performed in accordance with the Declaration of Helsinki. All patients gave written informed consent that comprised a data privacy clause for data collection and analysis for research purpose.

### Statistical Analysis

All statistical tests were performed using IBM SPSS Statistics Version 25. Associations between clinical baseline characteristics were evaluated using the Fisher’s exact chi- test. Kaplan-Meier method was used to analyze OS and PFS. The log-rank test was used to compare Kaplan-Meier survival curves. A p-value <0.05 was considered to be statistically significant. Cox proportional hazard methods were used to study the association between different baseline factors and OS. During the selection process, all explanatory factors with an effect p-value of <0.1 in the univariate Cox regression analysis were included in the multivariate analysis. A backward selection procedure was applied. Only the parameters with a p-value <0.05 in the backward selection procedure remained in the final model.

## Results

### Patient Characteristics

A total of 104 patients were registered for the ST-ICI trial between April 2017 and August 2019. Out of these, 50 patients received radiotherapy within a timeframe of ±30 days from the first administration of the immune checkpoint inhibitor and were included in this interim analysis. Baseline characteristics are presented in **Table 1**. Median age was 60 years (range, 37–87 years), 29 patients were male (58%). Radiotherapy of all metastases (al-RT) was performed in 27 (54%) patients, 23 (46%) patients received radiotherapy of only a single metastasis besides others (sl-RT). The mean number of metastases was 1.63 (95% CI, 1.34 – 1.92) in the al-RT group and 6.30 (95% CI, 4.72 – 7.89) in the sl-RT group (p < 0.001). Fourteen patients with one metastases were included in the al-RT group, whereas none in the sl-RT group (not possible). Most frequent tumor entity was non-small cell lung cancer (NSCLC) with 31 patients (62%), head and neck squamous cell cancer (HNSCC) in 13 patients (26%). Additional 3 patients with urothelial cancer (5%), one patient with esophageal cancer, one patient with mixed adenoneuroendocrine carcinoma in the gastric region and one patient with sinonasal-undifferentiated carcinoma were included in the analysis. PD-L1 expression was <1% in 15 patients (30%) and ≥1% in 33 patients (66). The majority of patients received prior local treatment. Previous treatment consisted of surgery of the primary tumor in 17 patients (34%) and radiotherapy in 39 patients (78%). In the recurrent or metastatic situation 35 patients (70%) received 1 to 2 lines of previous chemotherapy whereas 7 patients have received even more lines of chemotherapy. First line chemotherapy was platinum based in 36 patients (72%).

**Table 1:** Baseline Characteristics.

	No. (%) Patients			
Characteristic	Total (n = 50)	al-RT (n = 27)	sl-RT (n = 23)	p-value
Age, years				1.0
Mean +/- SD	60.9 ± 11.3	59.0 ± 10.5	63.3 ± 11.9	
Sex				0.398
Male	29 (58)	14 (51)	15 (65)	
Female	21 (42)	13 (49)	8 (35)	
Tumor entity				0.454
HNSCC	13 (26)	5 (18)	8 (34)	
NSCLC	31 (62)	18 (66)	13 (56)	
Urothelial cancer	3 (6)	2 (8)	1 (5)	
Others*	3 (6)	2 (8)	1 (5	
No. of Metastasis				<0.001
1	14 (28)	14 (52)	0	
2–5	25 (50)	13 (48)	12 (52)	
6–10	6 (12)	0	6 (26)	
>10	5 (10)	0	5 (22)	
CNS Metastases				0.136
No	34 (68)	21 (77)	13 (55)	
Yes	16 (32)	6 (23)	10 (45)	
No. Previous Chemotherapies in the recurrent/metastatic situation				1.0
0	12 (24)	6 (22)	6 (26)	
1–2	35 (70)	20 (74)	15 (65)	
>2	3 (8)	1 (4)	2 (9)	
No. of previous radiotherapies				0.367
0	4 (8)	3 (11)	1 (4)	
1–2	39 (78)	22 (81)	17 (74)	
>2	7 (14)	2 (8)	5 (22)	
1^st^ line Platin CT				0.251
Yes	36 (72)	21 (77)	15 (65)	
No	14 (28)	6 (23)	8 (35)	
Prior Surgery				0.239
Yes	17 (34)	7 (26)	10 (44)	
No	33 (66)	20 (74)	13 (56)	
PD-L1 Expression Tumor				0.215
<1%	15 (30)	6 (22)	9 (39)	
1–49%	12 (24)	9 (33)	3 (13)	
≥50%	21 (42)	11 (41)	10 (43)	
unknown	2 (4)	1(4)	1(5)	
Histology				0.167
Squamous Cell	19 (38)	9 (34)	10 (43)	
Adeno	20 (40)	14 (51)	6 (27)	
NSCLC NOS	5 (10)	1 (4)	4 (17)	
Urothelial	3 (6)	2 (7)	1 (5)	
Others**	3 (6)	1 (4)	2 (8)	

al-RT, all-lesion radiotherapy; sl-RT, single-lesion radiotherapy; SD, standard deviation; HNSCC, Head and Neck squamous cell carcinoma; NSCLC, non-small cell lung carcinoma; CNS, central nervous system; PD-L1, programmed cell death ligand 1.

*others include one case of esophageal cancer, on case of sinonasal undifferentiated cancer and one mixed adenoneuroendocrine carcinoma.

**Others include one case of mixed-neuroendocrine carcinoma (MANEC), one case of undifferentiated carcinoma (SNUC) and one case of acinic-cell-carcinoma.

There were no statistically significant differences of baseline characteristics in the al-RT and sl-RT cohort except the number of metastases.

### Treatment

Treatment characteristics are displayed in **Table 2**. The most frequent fractionation of radiotherapy was normal fractionation (single doses between 1.8 and 2.2 Gy) in 25 (50%) patients, followed by 22 (44%) patients receiving hypo-fractionation (single doses between 3 Gy and 6 Gy), three patients (6%) received radiosurgery (18 – 20 Gy). Radiotherapy was delivered to a single lesion in 14 patients (51%) of the al-RT group and 15 patients (65%) of the sl-RT group (p= 0.134). The location of radiotherapy differed significantly in both groups with the most frequent location being lung (51%) in the al-RT group and brain (39%) in the sl-RT group (p<0.001). Conventional fractionation was used more frequently in the al-RT group than in the sl-RT group (60% vs. 40%, p=0.023). The mean total administered dose was higher in the al-RT group than in the sl-RT group (55.3 Gy versus 43.4 Gy, p=0.004). In both cohorts the most frequently used immune checkpoint inhibitor was nivolumab (50%) followed by pembrolizumab (32%). Concomitant chemotherapy was administered in four patients (8%), whereas three were in the al-RT group and one in the sl-RT group

**Table 2:** Treatment characteristics.

	No. (%) Patients			
Treatment	Total (n = 50)	al-RT (n = 27)	sl-RT (n = 23)	p-value
Fractionation of radiotherapy				0.023
Normofract.	25 (50)	16 (60)	9 (40)	
Hypofract.	22 (44)	10 (37)	12 (52)	
SRS	3 (6)	1 (3)	2 (8)	
Total Dose				
Mean (SD)	49.8 ± 17.1 Gy	55.3 ± 16.6 Gy	43.4 ± 15.7 Gy	0.004
Median (range)	48 Gy (16–72)	60 Gy (18–72)	40 Gy (16–72)	
No. of irradiated lesions				0.134
1	29 (58)	14 (51)	15 (65)	
2	15 (30)	9 (34)	6 (26)	
>3*	6 (12)	4 (15)	2 (9)	
Location of radiotherapy				<0.001
Lung	17 (34)	14 (51)	3 (13)	
CNS	10 (20)	1 (5)	9 (39)	
Bone	5 (10)	0	5 (21)	
Other	18 (36)	12 (44)	6 (27)	
Drug				0.038
Nivolumab	25 (50)	11 (40)	14 (60)	
Pembrolizumab	16 (32)	9 (33)	7 (30)	
Other	9 (28)	7 (27)	2 (10)	
Concomitant Chemotherapy				0.614
Yes	4 (8)	3 (10)	1 (5)	
No	46 (92)	24 (90)	22 (95)	
irAE				0.121
any grade	15 (30)	11 (40)	4 (18)	
Grade 1–2	13 (26)	10 (37)	3 (13)	
Grade 3	2 (4)	1 (3)	1 (4)	

al-RT, all-lesion radiotherapy; sl-RT, single-lesion radiotherapy; SD, standard deviation; CNS, central nervous system; irAE, immune-related adverse event.

*1 patient irradiated on a total of 8 cerebral lesions.

### Safety

A total of 15 patients developed immune-related averse events (irAE). The rate of irAE seems increased in the al-RT group (al-RT n=11; sl-RT n=4). The predominant irAE was hypothyroidism (n=6), followed by skin reaction, hepatitis, diarrhea and pneumonitis that appeared in two patients each. The majority of these patients experienced CTCAE grade one or two toxicity (n=13), grade three was observed in two patients. The development of an irAE in a specific organ was not associated with the location of radiotherapy. No relevant radiation toxicity was reported within the follow-up time. Especially in patients with radiotherapy to brain metastases, no case of radionecrosis was reported until the most recent MRI.

### Efficacy

**Figure 1:**
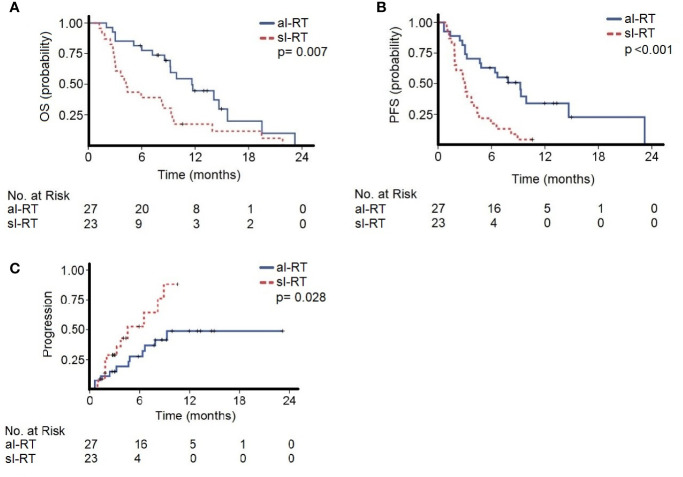
The cohorts with all-lesion radiotherapy (al-RT) and single-lesion radiotherapy (sl-RT) are compared regarding **(A)** overall survival (OS), **(B)** progression-free survival (PFS) and **(C)** time to progression (TTP).

The median follow-up of the entire cohort was 8.2 months (95% CI, 0.7–21.8) beginning with initiation of ICI. The median OS in the al-RT group was 11.6 months (95% CI, 8.1–15.1) and significantly longer than 4.2 months (95% CI, 3.0–5.4, p=0.007) in the sl-RT group (**Figure 1A**). After one-year OS was 30% in the al-RT group compared to 13% in the sl-RT group. The median PFS was 9.2 months (95% CI, 5.8 - 12.6) in the al-RT group and also significantly longer than 3.0 months (CI 95%, 2.5–3.5, p<0.001) in the sl-RT group (**Figure 1B**). The median TTP was not reached in the al-RT group compared to 4.6 months (95% CI, 1.1–8.0, p=0.028) in the sl-RT group (**Figure 1C**).

As the al-RT group contained patients with only one metastasis, which is not possible in the sl-RT group, additional Kaplan-Meier analyses were performed. In the al-RT group both OS and PFS were similar in patients with one metastasis of more metastases in the ST-ICI cohort The same accounts for Kaplan_Meier analyses within the sl-RT group (**Supplementary Figure 1**).

A univariate cox regression analysis was performed to study potentially prognostic factors for OS (**Table 3**). Tumor entity NSCLC, adenocarcinoma histology, absence of brain metastases, immunotherapy drug and treatment group al-RT were associated with lower mortality risk with an effect p-value of ≤ 0.1. These factors were included in the multivariable analysis. Age, sex, PD-L1 and number of previous treatments were not associated at a p-level of ≤ 0.1. In the multivariable analysis only tumor entity (NSCLC compared to HNSCC, HR 1.19, 95% CI, 0.46 – 3.10, p=0.028; other compared to HNSCC, HR 7.25, 95% CI, 1.49–35.28, p=0.028) and al-RT (HR 0.32, 95% CI, 0.14 – 0.75, p = 0.007) remained prognostic factors for overall survival.

**Table 3:** Univariate and multivariate Cox proportional hazard models to investigate the association between patient characteristics and overall survival.

Explanatory factors	(*N = 50*)	Univariate					Multivariate^#^		
		N	Death	HR	95% CI	p-value	HR	95% CI	p-value
Age*	≤60 years	16	11	1		0.748			
	>60 years	34	21	1.12	0.53–2.37				
Sex*	Male	29	21	1		0.833			
	Female	21	11	1.08	0.50–2.334				
Tumor entity	HNSCC	13	10	1		0.064	1		0.028
	NSCLC	31	17	0.42	0.18–0.97		1.19	0.46 - 3.10	
	other	6	5	1.13	0.38–3.31		7.25	1.49 - 35.28	
Histology	SC	19	14	1		0.011			
	Adeno	20	9	0.27	0.10–0.70				
	other	11	9	0.81	0.34–1.92				
PD-L1 tumor cells*	<1%	15	13	1		0.491			
	1–49%	12	6	0.67	0.24–1.82				
	≥50%	21	12	0.62	0.28–1.39				
Brain metastases	no	16	13	1		0.089			
	yes	34	19	1.89	0.92–3.89				
Number of previous treatments*	0–1	32	16	1		0.265			
	≥2	18	16	1.5	0.74–3.05				
Immunotherapy drug	Nivo	25	21	1		0.064			
	Pembro	16	8	0.49	0.22–1.14				
	other	9	3	0.32	0.09–1.09				
Lesions irradiated	al-RT	27	13	1		0.005	1		0.007
	sl-RT	23	19	0.36	0.17–0.74		0.32	0.14 - 0.75	

HR, hazard ratio; CI, confidence interval; HNSCC, head and neck squamous cell cancer; NSCLC, non-small cell lung cancer; SC, squamous cell; Nivo, nivolumab; Pembro, pembrolizumab; al-RT, all-lesion radiotherapy; sl-RT, single-lesion radiotherapy.

*Within the selection process, only explanatory factors with an effect p-value of < 0.1 in the univariate Cox regression analysis were considered (*).

^#^Final Cox regression model after backward selection. Only factors with p < 0.05 remained in the final model.

## Discussion

Local ablative treatments in an oligometastatic situation gained importance during the past years (1). Particularly in oligometastatic lung cancer the addition of local therapy of all tumor lesions in patients responding to systemic chemotherapy improved OS (3). Recent studies investigated especially the role of stereotactic radiotherapy as local ablative treatment method and found a clinical benefit (15). Furthermore, in a phase II trial a survival benefit for local consolidative radiotherapy of the primary tumor in metastatic NSCLC was also found (11). These studies justify the addition of local treatments to all lesions to classical chemotherapy in patients with oligometastatic cancer.

During the past years, immune checkpoint inhibitors suppressing the PD-1/PD-L1 pathway improved prognosis in different tumor entities significantly and became first line treatment in metastatic melanoma, NSCLC, HNSCC, renal and bladder cancer (4, 5, 16–18). In this new first line treatment setting, the efficacy of treatment to all known metastatic sites has not been studied so far.

In this regard, the ST-ICI study investigates the effects of radiotherapy in combination with PD-1/PD-L1 immune checkpoint inhibitors. The presented analysis shows that patients with oligo-metastatic disease treated with al-RT had superior OS, PFS and TTP compared to patients with sl-RT. This advocates a benefit of local therapy to all tumor lesions also in the era of immune checkpoint inhibitors. In general, the combination of radiotherapy and immunotherapy in the ST-ICI trial seems not increase the frequency or severity of irAEs as they were similar to reports on SCLC and HSNCC treated with anti-PD-1 monotherapy (4, 5). However, the frequency of irAE seems increased in the al-RT compared to the sl-RT cohort. Previous analyses of concomitant radiotherapy and immunotherapy indicated increased toxicity (19). The increased rate of irAE may be partially explained by immune modulating effects of radiotherapy resulting in the activation of tumor-surrounding immune cells (20).

Limitations of the study are based on the inevitable shortcomings due to the missing randomization. Thus, the results of the study need to be interpreted in view of the different tumor entities, number of metastases and minor differences in the PD-L1 status. These partially differing baseline characteristics may have influenced the results of this analysis. A major limitation is the differing number of metastases in both treatment groups. Due to this imbalance additional Kaplan-Meier analyses were performed. Both in the al-RT and sl-RT group the number of metastases did not influence PFS or OS. Regarding radiotherapy treatment the location, dose and fractionation of the two groups differed. Especially the more frequent irradiation of brain metastases in the sl-RT group and lung metastases in the al-RT group may be a bias for survival. However, al-RT was the most important predictor of OS in the multivariate model, which consolidates the findings of this trial. Especially brain metastases remained no prognostic parameter in the multivariate model. The main advantage of the ST-ICI trial is its prospective design compared to existing analyses.

Prospective data about the combination of radiotherapy and immunotherapy in oligometastatic situations are limited. In a recent single-arm phase two trial patients with oligometastatic NSCLC (up to four metastases) were treated with local ablative treatment (radiotherapy or surgery) followed by pembrolizumab. There was no limitation regarding PD-L1 status. This strategy achieved an impressive median PFS of 19.1 months (21). One analysis studied treatment continuation with pembrolizumab beyond progression in NSCLC patients with PDL1>50% with the addition of local therapy in nine of 18 patients and found a one-year OS of 71% (22).

Besides these clinical findings, there is a strong biological rationale to combine radiotherapy with immune checkpoint inhibitors. Radiotherapy not only kills tumor cells, but also has different immune-modulating effects (7). Locally induced immune-modulating effects of radiotherapy can also enhance tumor directed systemic immune response distant from the irradiated area (10). Mechanisms behind these effects are increase in T-cell infiltration in locally treated tumors and further enhanced T-cell responses out of field (23). These systemic immune responses to local radiotherapy, also called “abscopal effects”, have been observed in patients with combined radiotherapy and immune checkpoint inhibitors in numerous case reports (24). Additional retrospective analyses indicated the presence of abscopal effects in up to 25% of melanoma patients treated with PD-1 inhibitors and radiotherapy (25). However, in a prospective single arm trial the combination of Nivolumab with stereotactic body radiation therapy of a single tumor lesion did not increase the response rate in melanoma patients compared to historical controls (26). A trial in metastatic head and neck cancer that combined nivolumab with single lesion stereotactic radiotherapy did also not increase the response rate in non-irradiated lesions (27). The only partially promising prospective trial was in metastatic NSCLC. In this trial stereotactic radiotherapy increased the response rate in non-irradiated lesions from 18% to 36%, whereas this did not reach statistical significance (28). In contrast to these trials, our trial compared the efficacy of single lesion radiotherapy in patients with multiple metastases to radiotherapy of all tumor lesions. Furthermore, we used fractionated radiotherapy compared to stereotactic ablative approaches in the other trials. In our study radiotherapy of all tumor lesions significantly prolonged overall survival. Recently, also a correlation of tumor burden and reduced immune competence was demonstrated (29). Therefore, a maximal reduction of tumor load appears to be beneficial for the effect of immunotherapy. Our findings are in line with another prospective single-arm trial in metastatic NSCLC of stereotactic radiotherapy to all tumor lesions followed by Pembrolizumab maintenance therapy that achieved a median overall survival of 41.6 months (21). The results of these first prospective trials on abscopal effects should induce a discussion on more elaborate treatment concepts (30). In this discussion, our finding of a superiority of radiotherapy to all lesions compared to a single lesion should be addressed. Furthermore, the fractionation of radiotherapy with either conventionally fractionated low doses or high ablative doses has to be discussed. In addition, the treatment sequence of concomitant versus sequential administration of PD-1 inhibitors should be further investigated. And finally, also a biomarker-based patient selection should be an essential point in future trials on combined radio-immunotherapy.

## Data Availability Statement

The raw data supporting the conclusions of this article will be made available by the authors on reasonable request, without undue reservation.

## Ethics Statement

The studies involving human participants were reviewed and approved by the ethics committee of Friedrich-Alexander University Erlangen Nürnberg (number: 2_17 B). The patients/participants provided their written informed consent to participate in this study.

## Author Contributions

Conception/design: PS, MHe, UG, RF. Collection and/or assembly of data: PS, SR, ME, CS, MHe, A-OG, SS, MHa, SL. Data analysis and interpretation: PS, J-GZ, MHe, BF, UG, RF. Manuscript writing: PS, A-OG, MHe. Final approval of manuscript: PS, SR, ME, BF, CS, MHa, SL, SS, A-OG, J-GZ, UG, RF, MHe. All authors contributed to the article and approved the submitted version.

## Conflict of Interest

SR conflict of interest with AstraZeneca (research funding); MSD (research funding). ME conflict of interest with Diaceutics (employment, honoraria, advisory role, speakers’ bureau, travel expenses); AstraZeneca (honoraria, advisory role, speakers’ bureau, travel expenses); Roche (honoraria, travel expenses); MSD (honoraria, speakers’ bureau); GenomicHealth (honoraria, advisory role, speakers bureau, travel expenses); Astellas (honoraria, speakers’ bureau); Janssen-Cilag (honoraria, advisory role, research funding, travel expenses); Stratifyer (research funding, patents). SS conflict of interest with Strycker (stock); Varian (stock); Abbot (stock); Crispr Techn. (stock); Pfitzer (stock); Merck Serono (stock); Symrise (stock); Ortho (honoraria, advisory role, speakers’ bureau, research funding, travel expenses); PharmaMar (speakers’ bureau, travel expenses); Haema (speakers’ bureau). UG conflict of interest with AstraZeneca (advisory role, research funding); BMS (advisory role); MSD (research funding); Sennewald Medizintechnik (travel expenses). RF conflict of interest with MSD (honoraria, advisory role, research funding, travel expenses); Fresenius (honoraria); BrainLab (honoraria); AstraZeneca (honoraria, advisory role, research funding, travel expenses); Merck Serono (advisory role, research funding, travel expenses); Novocure (advisory role, speakers’ bureau, research funding); Sennewald (speakers’ bureau, travel expenses). MHe conflict of interest with Merck Serono (advisory role, speakers’ bureau, honoraria, travel expenses, research funding); MSD (advisory role, speakers’ bureau, travel expenses, research funding); AstraZeneca (research funding); Novartis (research funding); BMS (advisory role, honoraria, speakers’ bureau); Teva (travel expenses).

The remaining authors declare that the research was conducted in the absence of any commercial or financial relationships that could be construed as a potential conflict of interest.

## Supplementary Material

Supplementary Figure 1 The numbers of metastases of the cohorts all-lesion radiotherapy (al-RT) are compared regarding **(A)** overall survival (OS) and **(B)** progression-free survival (PFS). Patients of the single-lesion radiotherapy cohort (sl-RT) were compared concerning **(C)** overall-survival (OS) and **(D)** progression-free-survival (PFS).
