# Supplementary figures and images for: Prospective Evaluation of All-lesion Versus Single-lesion Radiotherapy in Combination With PD-1/PD-L1 Immune Checkpoint Inhibitors
Source: Front Oncol. 2020 Oct 29;10:576643. doi: 10.3389/fonc.2020.576643 (PMC7673414; doi:10.3389/fonc.2020.576643)

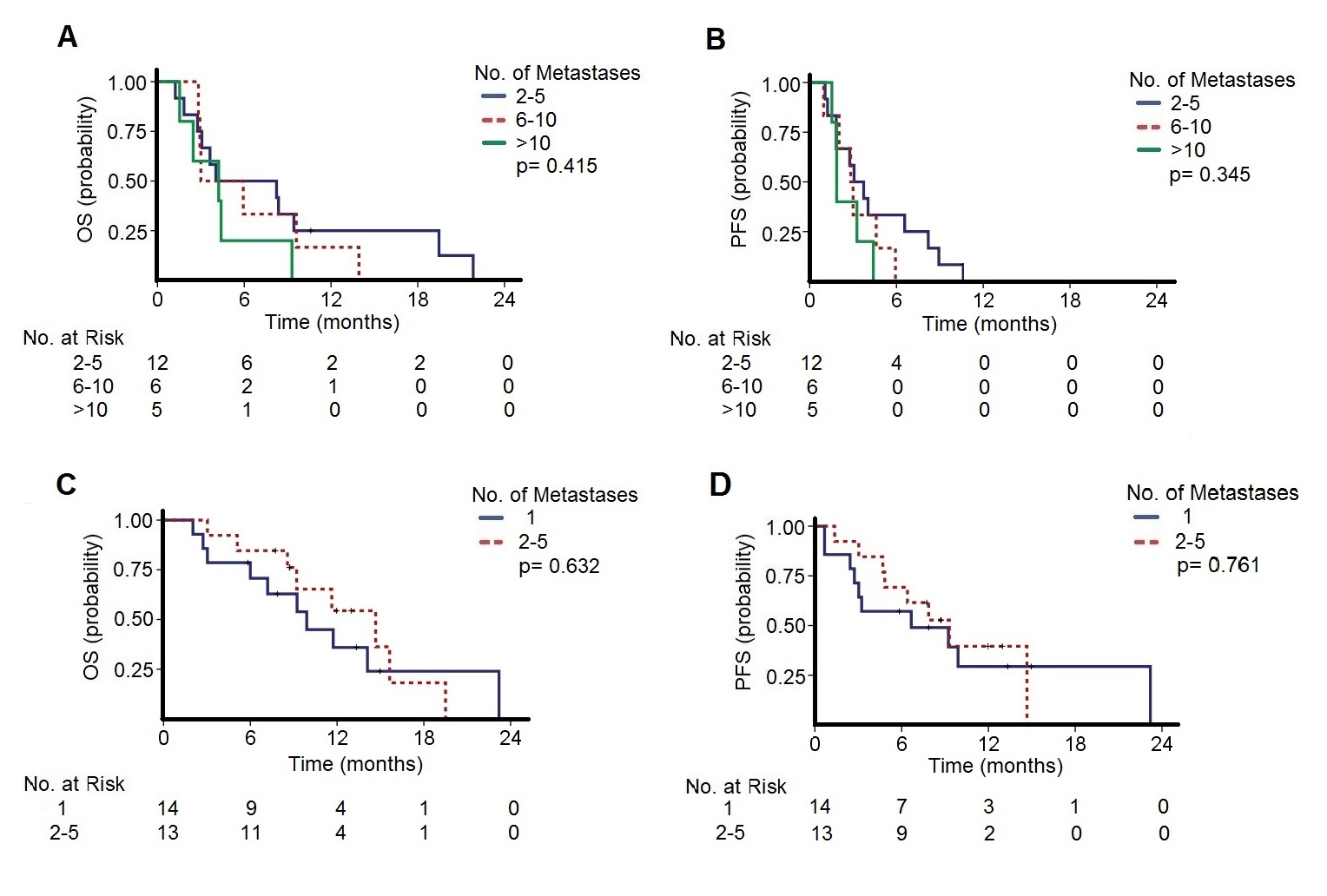

Supplement: Supplementary Figure 1 — The numbers of metastases of the cohorts all-lesion radiotherapy (al-RT) are compared regarding (A) overall survival (OS) and (B) progression-free survival (PFS). Patients of the single-lesion radiotherapy cohort (sl-RT) were compared concerning (C) overall-survival (OS) and (D) progression-free-survival (PFS). [file Image_1.tif]
